# Clinical features and outcome of acute hepatitis B in pregnancy

**DOI:** 10.1186/1471-2334-14-368

**Published:** 2014-07-03

**Authors:** Yong-Tao Han, Chao Sun, Cai-Xia Liu, Shuang-Shuang Xie, Di Xiao, Li Liu, Jin-Hong Yu, Wen-Wen Li, Qiang Li

**Affiliations:** 1 Department of Pharmacy, Qilu Hospital, Shandong University, Jinan, China; 2 Division of Liver Disease, Jinan Infectious Disease Hospital, Shandong University, Jinan, China; 3 Unit of Disease Control Genome Medicine, Institute of Medical Science, University of Tokyo, Tokyo, Japan

**Keywords:** Acute hepatitis B, Pregnancy, Clinical features, Outcome, Hepatitis B surface antigen, Chronicity

## Abstract

**Background:**

The impact of pregnancy on the clinical course of acute hepatitis B (AHB) is still largely unclear, mainly because most studies have not included matched controls. This study was conducted to investigate the clinical features and outcome of AHB in pregnancy using matched controls.

**Methods:**

Consecutive AHB inpatients who were admitted to Jinan Infectious Disease Hospital, Jinan, between January 2006 and December 2010 were evaluated and followed. Demographic data, clinical manifestations, and results of laboratory tests were compared between pregnant patients and age and sex matched non-pregnant patients at admission, discharge, and final follow-up.

**Results:**

A total of 618 AHB inpatients were identified during the study period. 22 pregnant patients and 87 age and sex matched non-pregnant patients were enrolled in this study. Prodromal fever was less common (0% vs. 20.7%, *P* = 0.02), serum alanine aminotransferase levels were significantly lower, and HBsAg > 250 IU/mL rate and serum bilirubin levels were significantly higher in pregnant patients than in non-pregnant patients. After a mean (range) of 7(5.2-8.3) months follow-up, 18.2% pregnant patients and 4.6% non-pregnant patients were still HBsAg positive (*P* = 0.03). For pregnant patients, the relative risk (95% confidence interval) of HBsAg positive at the end of follow-up was 4.6 (1.1-20.2). The median (95% confidence interval) days of HBsAg seroclearance form disease onset in pregnant and non-pregnant patients were 145.0 (110.5-179.5) and 80.0 (62.6-97.4), respectively.

**Conclusions:**

The HBsAg loss and seroconversion were delayed and lower in pregnant patients. Pregnancy might be a possible risk of chronicity following acute HBV infection.

## Background

It is well-known that acute hepatitis B virus (HBV) infection in adults is usually self-limiting, of which only 5%-10% develop to chronic HBV infection, and about 1% progress to acute liver failure [1]. However, in certain special populations, such as pregnant women, the clinical course of acute hepatitis B (AHB) is still largely unclear. Although a few studies have investigated the impact of pregnancy on AHB, few of them included matched controls [2-8]. Age, sex, and host immunity status are important factors influencing the clinical manifestation and outcome of acute HBV infection [9]. Clinical features and HBV serological outcome in pregnant AHB patients, such as hepatitis B surface antigen (HBsAg) loss and seroconversion, have not fully been elucidated.

The clinical presentation and natural history of HBV infection is mediated through complex interactions between the virus and the host immune response [10]. In acute HBV infection, both host innate immunity and HBV specific adaptive immunity play important roles in viral clearance [10,11]. Impaired immunity due to various causes may influence the course of AHB [9,12-14]. During pregnancy, the maternal immune system is altered to tolerate the genetically different fetus, and hormonal factors may also play a significant role in altering immune regulation or viral replication [15]. An early study by Mohite et al. reported that the T cell response to HBsAg was weaker in pregnant women than in adults males and non-pregnant females [16]. So, immunological response to acute HBV infection, and consequently the clinical course of AHB in pregnant women may differ from those in the general population [16].

The aim of this study was to investigate the clinical features and outcome of AHB in pregnancy.

## Methods

### Study design and population

The present study was performed in the Jinan Infectious Disease Hospital, the only hospital in the Jinan area which is in charge of the diagnosis and treatment of the 35 notifiable infectious diseases of China. This study is part of a hospital-based prospective investigation to study the natural history of acute HBV infection that was conducted in the hospital,in which all inpatients with confirmed AHB between January 2006 and December 2010 were included and followed. The study was approved by the hospital ethics committee, and written informed consent for participation was obtained from each study participant or their next of kins.

The diagnosis of AHB was based on discrete onset of symptoms (such as: fever, loss of appetite, fatigue, and dark urine), jaundice, elevated serum alanine aminotransferase (ALT) or aspartate aminotransferase (AST) levels, detection of high-titer IgM antibody to hepatitis core antigen (anti-HBc), and compatible clinical history [17,18].

Patients with a history or other evidence of liver disease other than AHB, or with alcohol consumption of more than 20 g/day for more than five years were excluded. All patients were to be followed for at least 6 months. In general, follow-up schedule was 1 month, 3 months, and 6 months after discharge.

In this study, the clinical manifestation, biochemical and virological parameters, and outcome of pregnant AHB patients were compared with those of sex and age matched non-pregnant AHB patients.

### Study variables

The variables analyzed in this study included demographic data, prodromal signs and symptoms, complications, serum biochemical parameters, HBV serological marker, HBV DNA levels at admission, discharge and final follow-up. Days from disease onset to patient’s hospital admission, duration of hospitalization and follow-up were also analyzed.

Upon entry to the hospital, all patients were interviewed by trained physicians. The details of information collection and laboratory measurements were previously described [19,20]. The transmission routes and risk factors of HBV infection were recorded using a structured questionnaire. In general, biochemical parameters, HBV serological markers, and HBV DNA levels, were measured at least once at admission, before discharge, and at each visit during follow-up. A form designed for this investigation was used to record the information collected at every visit.

For patients with jaundice, prodromal manifestations were defined as signs or symptoms occurring in the 1–10 days before onset of icterus (prodromal period). For patients without jaundice, prodromal manifestations were defined as signs or symptoms occurring in the 1–10 days before diagnosis of hepatitis or the hospital admission.

Prodromal signs and symptoms analyzed in this study included fever, loss of appetite, fatigue. Prodromal fever were defined in 2 different ways: patients reporting a measured fever ≥ 37.5°C during prodromal period; any fever (measured or subjective) during prodromal period [21,22]. Jaundice referred to measured serum total bilirubin > 25 μmol/L at admission. HE was diagnosed and classified as previously described [23]. The presence of ascites was evaluated using B ultrasonography.

Clinical outcome included occurrence of fulminant hepatitis, clinical recovery (defined as relief of signs and symptoms and normalization of ALT levels), and mortality. HBV virological and serological outcome included undetectable HBV DNA, HBsAg loss, HBsAg seroconversion (i.e. loss of HBsAg and appearance of anti-HBsAg antibodies), hepatitis B e antigen (HBeAg) seroconversion (i.e. loss of HBeAg and appearance of anti-HBeAg antibodies). Chronic HBV infection was defined as persistent of HBsAg in serum with or without detectable serum HBV DNA after disease onset of more than 6 months [24]. AHB with evidence of coagulation abnormality (INR ≥ 1.5) and encephalopathy was diagnosed as acute fulminant hepatitis [23].

### Statistical analysis

Clinical parameters were evaluated using the chi-squared test for discrete variables and the t test or Mann–Whitney U test for continuous variables. A Kaplan–Meier estimate was performed to compare the fraction of patients remain HBsAg positive and days of HBsAg seroclearance after disease onset, and p values were calculated using log-rank test. For all tests, a *P* value of less than 0.05 was considered significant. All data analyses were performed using SPSS v. 16.0 (SPSS Inc., Chicago, IL, USA).

## Results

Among a total of 618 inpatients diagnosed with AHB during the study period, 22 pregnant patients were enrolled into the pregnant group. The mean ± SD and median (min-max) age of the AHB in pregnant patients was 22.7 ± 1.9 years and 22 (21–28) years, respectively. 87 female non-pregnant patients between 21 and 28 years of age were enrolled in the control group.

### Demographic characteristics of AHB in pregnancy

The demographic characteristics of the two groups are summarized in Table 1. Most pregnant AHB patients were from rural areas of Shandong Province (90.9%). The mean length from onset of signs and symptoms to hospital admission was similar between the two groups. Of the 22 pregnant AHB patients, 8 were in the first trimester, 8 were in the second trimester, and 6 were in the third trimester of pregnancy. The mean length (min-max) of pregnancy was 17.3 (7–31) weeks. 72.5% of the patients ever exposed to at least one potential risk factor of acute HBV infection. No pregnant patients had gestational diabetes mellitus.

**Table 1:** Demographics and the risk factors exposed within 6 months before onset of AHB: comparison of pregnant and non-pregnant patients

**Variables**	**Non-pregnant AHB patients N = 87**	**Pregnant AHB patients N = 22**	***P***
**Age (years)**	23.3 ± 2.6	22.7 ± 1.9	0.18
**Residence**			0.001
Urban	42 (48.3%)	2 (9.1%)	
Rural	45 (51.7%)	20 (90.9%)	
**Days from disease onset to hospital admission**	10.9 ± 7.5	10.6 ± 4.1	0.78
**Days of stay in hospital**	28.7 ± 10.3	24.7 ± 7.4	0.09
**Weeks of pregnancy**	NA	17.3 ± 8.7	NA
**Potential risk factors**			0.17
Spouse with HBV infection	8 (9.2%)	6 (27.3%)	
Other family members with HBV infection	3 (3.4%)	1 (4.5%)	
Risk sex behavior	32 (36.8%)	3 (13.6%)	
Invasive medical procedure	12 (13.8%)	4 (18.2%)	
Body care	8 (9.2%)	2 (9.1%)	
Unknown	24 (27.6%)	6 (27.3%)	
More than one factor	35 (55.6%)	7 (45.7%)	0.40

NOTE. Data are expressed as mean ± SD or n (%) of patients; AHB, acute hepatitis B.

### Clinical and biochemical characteristics of AHB in pregnancy

As shown in Table 2, fever was less frequent, and jaundice was more frequent in pregnant patients than in non-pregnant patients.

**Table 2:** Symptoms,signs, and complications of pregnant and non-pregnant AHB patients

**Variables**	**Non-pregnant AHB patients N = 87 (%)**	**Pregnant AHB patients N = 22 (%)**	***P***
Fever	18 (20.7)	0 (0)	0.02
Fatigue	65 (74.7)	15 (68.2)	0.54
Loss of appetite	70 (80.5)	14 (63.6)	0.08
Jaundice	56 (62.2)	19 (95.4)	0.003
Ascites	1 (1.15)	0 (0)	0.61
Encephalopathy	1 (1.15)	0 (0)	0.61

NOTE. AHB, acute hepatitis B.

The biochemical data of pregnant and non-pregnant AHB patients at admission, discharge, and final follow-up are summarized in Table 3. At admission, the median ALT levels of pregnant patients were significantly lower than that of non-pregnant patients. The median total serum bilirubin level was higher in pregnant patients than in non-pregnant patients. The median serum albumin and gamma-glutmyltransferase (GGT) levels were both significantly lower in pregnant patients than in non-pregnant patients (*P* < 0.001).

**Table 3:** Biochemical data in pregnant and non-pregnant AHB patients at admission, discharge and final follow-up

**Variables**	**Non-pregnant AHB patients N = 87**	**Pregnant AHB patients N = 22**	***P***
**At admission**			
Days from disease onset to examination	11.8 ± 7.3	11.3 ± 4.1	0.70
ALT(IU/L)	1237 (274–3615)	1025 (285–1797)	0.039
ALT > 1600 IU/L	27 (31.0%)	1 (4.5%)	0.01
Bilirubin(μmol/L)	66.4 (8.2-549.7)	140 (29.6-289.1)	0.005
Bilirubin > 25 μmol/L	69 (79.3%)	22 (100%)	0.02
Albumin(g/L)	39.0 (28–66)	33.0 (23.0-42.3)	< 0.001
Albumin < 35 g/L	12 (13.8%)	14 (63.6%)	< 0.001
GGT(IU/L)	95 (22–425)	32.5 (21–159)	< 0.001
GGT > 64 IU/L	68 (78.1%)	4 (18.2%)	< 0.001
**At discharge**			
Days from disease onset to examination	38.6 ± 11.9	34.2 ± 7.1	0.11
ALT(IU/L)	28 (7–712)	17 (9–128)	0.004
ALT normalization	56 (64.4%)	20 (90.9%)	0.02
Bilirubin(μmol/L)	15.2 (4.2-212.5)	19.7 (8–61.4)	0.27
Bilirubin normalization	65 (74.7%)	17 (77.3%)	0.80
Albumin(g/L)	40.4 (32–48.4)	35 (30–38.8)	< 0.001
Albumin normalization	85 (97.7%)	12 (54.5%)	< 0.001
GGT(IU/L)	48.0 (10–227)	28.5 (12–93)	< 0.001
GGT normalization	60 (69.0%)	20 (90.9%)	0.04
**Final follow-up**			
Months from disease onset to examination	7.0 ± 0.8	6.9 ± 0.7	0.54
ALT(IU/L)	20 (5–68)	12 (6–37)	0.015
ALT normalization	83 (95.4%)	22 (100%)	0.31
Bilirubin(μmol/L)	12.1 (4.2-56)	13 (5–22)	0.47
Bilirubin normalization	83 (94.4%)	22 (100%)	0.31
Albumin(g/L)	43 (36–48.5)	39.5 (34–43.0)	< 0.001
Albumin < 35 g/L	0 (0%)	3 (13.6%)	< 0.001
GGT(IU/L)	37 (10–87)	20 (12–30)	< 0.001
GGT normalization	80 (91.9%)	22 (100%)	0.17

NOTE. Data are expressed as mean ± SD, median(min-max), or n (%) of patients. AHB, acute hepatitis B; ALT, alanine aminotransferase; GGT, gamma-glutmyltransferase.

After a mean (min-max) of 27.8 (5–69) day’s supportive treatment, clinical manifestations and biochemical data of all patients were significantly improved.

After a mean (min-max) of 7 (5.2-8.3) months follow-up, ALT and bilirubin normalization rate were similar between the two groups. However, the median ALT, serum albumin and GGT levels were still significantly lower in pregnant patients than in non-pregnant patients.

### HBV virological and serological outcome of AHB in pregnancy

The HBV virological and serological biochemical data of pregnant and non-pregnant AHB patients at admission, discharge, and final follow-up are summarized in Table 4. At admission, the median HBV DNA level was similar between the two groups (*P* = 0.69), and the HBV DNA > 1000 copies/mL rate was also similar between the two groups. The HBsAg positive (>0.05 IU/mL) rate was similar between the two groups. The HBsAg > 250 IU/mL rate and HBeAg positive rate were significantly higher in pregnant than in non-pregnant patients.

**Table 4:** HBV serological and virological data in pregnant and non-pregnant AHB patients at admission, discharge and final follow-up

**Variables**	**Non-pregnant AHB patients N = 87**	**Pregnant AHB patients N = 22**	***P***
**At admission**			
Days from disease onset to examination	11.8 ± 7.3	11.3 ± 4.1	0.70
HBsAg positive	6 (93.1%)	22 (100%)	0.21
HBsAg > 250 IU/mL	45 (51.7%)	17 (77.3%)	0.03
HBsAg seroconversion	3 (3.44%)	0 (0%)	0.38
HBeAg positive	52 (59.8%)	19 (86.4%)	0.02
HBeAg seroconversion	33 (37.9%)	2 (9.1%)	0.01
HBV DNA (log_10_ copies/mL)	3.49 (3.0-7.14)	3.49 (3.0-4.93)	0.69
HBV DNA >1000 copies/mL	55 (63.2%)	17 (77.3%)	0.21
**At discharge**			
Days from disease onset to examination	38.6 ± 11.9	34.2 ± 7.1	0.11
HBsAg positive	54 (62.1%)	19 (86.4%)	0.03
HBsAg > 250 IU/mL	19 (21.8%)	9 (40.9%)	0.07
HBsAg seroconversion	11 (12.6%)	0 (0%)	0.08
HBeAg positive	8 (9.2%)	3 (13.6%)	0.54
HBeAg seroconversion	72 (82.8%)	15 (68.2%)	0.13
HBV DNA >1000 copies/mL	11 (12.6%)	1 (4.5%)	0.28
**Final follow-up**			
Months from disease onset to examination	7.0 ± 0.8 (5.2-8.3)	6.9 ± 0.7 (5.8-8.2)	0.54
HBsAg positive	4 (4.6%)	4 (18.2%)	0.03
HBsAg > 250 IU/mL	1 (1.1%)	1 (4.5%)	0.29
HBsAg seroconversion	59 (67.8%)	9 (40.9%)	0.02
HBeAg positive	1 (1.1%)	1 (4.5%)	0.29
HBeAg seroconversion	80 (91.9%)	19 (86.3%)	0.42
HBV DNA > 1000 copies/mL	1 (1.1%)	1 (4.5%)	0.29

NOTE. Data are expressed as mean ± SD,median (min-max), or n (%) of patients; HBV, hepatitis B virus; AHB, acute hepatitis B; HBsAg,hepatitis B surface antigen; HBeAg, hepatitis e antigen.

**Figure 1:**
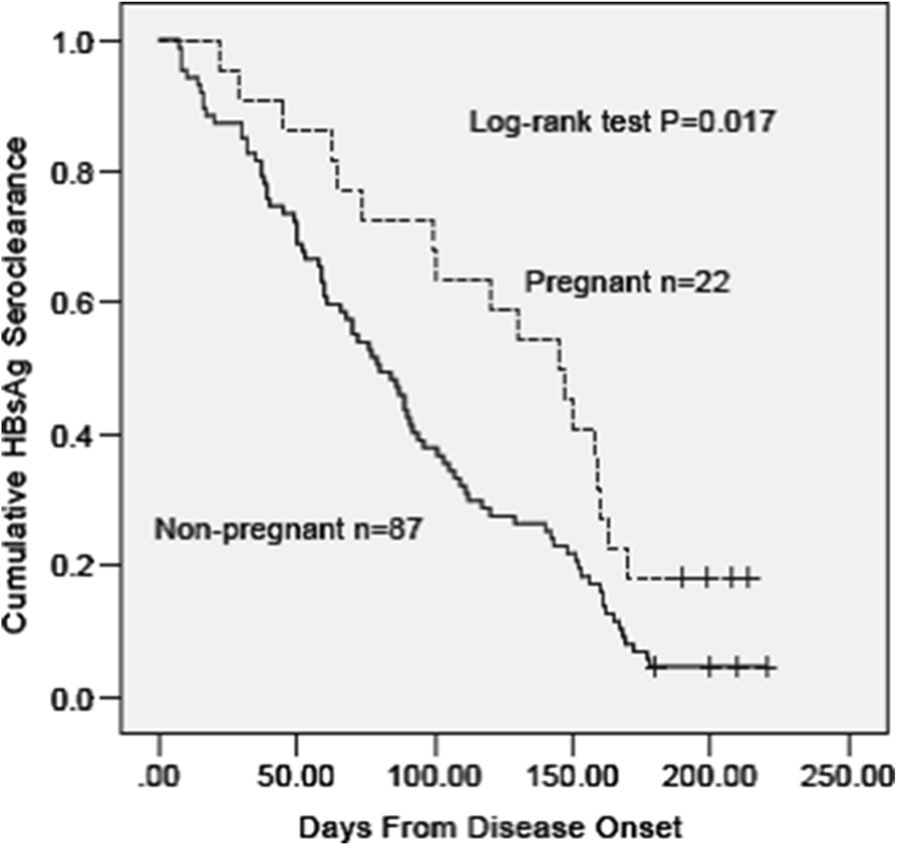
**Changes of the HBsAg status after disease onset.** Cumulative rates of HBsAg seroclearance, analyzed with the Kaplan–Meier test.

At discharge, the HBsAg positive rate was significantly higher in pregnant patients than in non-pregnant patients, whereas virological and other serological parameters were similar between the two groups.At final follow-up, the HBsAg positive rate was significantly higher in pregnant patients than in non-pregnant patients. 18.2% pregnant patients and 4.6% non-pregnant patients developed chronic HBV infection. For pregnant patients, the relative risk (95% confidence interval) of HBsAg positive at the end of follow-up was 4.6 (1.1-20.2). The Kaplan–Meier estimates showed the median (95% confidence interval) days of HBsAg seroclearance form disease onset in pregnant and non-pregnant patients were 145.0 (110.5-179.5) and 80.0 (62.6-97.4), respectively (Figure 1).

## Discussion

Just as it is generally accepted that pregnancy does not alter clinical recovery and mortality of AHB [25], the present study also did not find any significance difference on AHB mortality and occurrence of flulminant hepatitis between pregnant and non-pregnant patients. Clinical recovery, defined as relief of signs and symptoms, and normalization of ALT, was also similar between pregnant and non-pregnant females. However, the present study did reveal certain clinical and serological features of AHB during pregnancy.

Most of the patients in the present study were symptomatic (83.8%), whose signs and symptoms included prodromal fever, fatigue, and loss of appetite. Fever was significantly less common (0% vs. 20.7%, *P* = 0.02) in pregnant than in non-pregnant AHB patients, whereas the presence of other symptoms, such as fatigue and loss of appetite, were similar between the two groups. This finding may be partly explained by the “febrile hyporesponsiveness” phenomenon during pregnancy [26]. The febrile response is a significant contributor to the pathogenesis, clinical presentation and outcome of many illnesses and diseases [21]. During pregnancy, febrile response is suppressed by the following potential mechanisms: suppression of pro-inflammatory processes, augmentation of anti-inflammatory processes, and alterations in steroid hormones [21]. The suppressed febrile response during pregnancy can lead to a compromised ability to fight infection while it could be beneficial to the mother and the fetus. Whether the altered immunity during pregnancy affects HBV DNA replication and eradication is still inconclusive.

Several studies investigated the effect of pregnancy on serum HBV DNA levels of pregnant women with chronic HBV infection [27-29]. The study by Soderstrom et al. observed increased serum HBV DNA levels in late pregnancy or early post-partum [28]. But, Nguyen et al. reported that the levels of HBV DNA and ALT during pregnancy were highly variable [27]. Little information is available regarding the virological and serological changes during acute HBV infection in pregnant patients.

In the present study, the median HBV DNA levels and HBV DNA undetectable rate were similar between pregnant and non-pregnant patients. HBV DNA and ALT levels were highly variable in both groups.

We noted significant lower ALT levels in pregnant women. A study on acute liver failure during pregnancy by Bhatia et al. also observed significantly lower ALT levels in pregnant women than in men and non-pregnant women [30]. Elevated serum ALT levels or symptoms reflect the T-cell-mediated HBV-specific immune response [10]. The mild symptoms and relatively low ALT levels in pregnant AHB patients indicate that HBV specific immunity in pregnancy may differ from those in the general population.

Interestingly, we noted a significantly higher HBsAg levels and lower HBsAg severconversion rate in pregnant patients than in non-pregnant patients. At admission and discharge, the percent of HBsAg > 250 IU/mL in pregnant patients was higher than that in non-pregnant patients. Moreover, at final follow-up, about 7 months from disease onset, 18.2% of the pregnant patients were still HBsAg positive, a chronic HBsAg carrier state, whereas only 4.6% of the non-pregnant patients were HBsAg positive. Also, at final follow-up, the HBsAg seroconversion rate was significantly lower in pregnant than in non-pregnant patients (40.9% vs. 67.8%, *P* = 0.02). HBsAg levels reflect the host immune control state and cccDNA levels in hepatocytes [31,32]. T-cells are markedly reduced during early pregnancy up to the 20th week of gestation to reduce the level of immunity [15]. Increased hormone levels during pregnancy, including progesterone, estrogen and human chorionic gonadotropin, have been shown to have a clear suppressive effect on cell-mediated immunity [26]. This status may indicate that the altered immunity status may influence the eradication of HBV infected cells and the production of HBsAg antibodies. This notion is supported by the study of Mohite et al. which reported that the T cell response to HBsAg in pregnant women with AHB was absent throughout the illness, whereas that in adult males and non-pregnant females increased gradually after AHB onset [16].

We also noted a significantly more frequent and severe jaundice in pregnant women. At admission, hyperbilirubinemia was seen in all pregnant patients, and the total bilirubin level was significantly higher in pregnant than in non-pregnant patients. In acute hepatitis, serum total bilirubin level usually correlates positively with severity of liver injury [33]. However, we observed an inconsistence of total bilirubin level with peak ALT levels, a sensitive indicator of liver injury, in pregnant AHB patients. This indicate hyperbilirubinemia in pregnant AHB patients might be caused not only by intrahepatic inflammation, but possibly also by other factors influencing bilirubin clearance. Serum albumin and UDP-glucuronosyltransferase(UGP) are involved in bilirubin metabolism [34,35]. Significant lower serum albumin level, a protein that transports bilirubin, were observed in our and other studies [34,36]. An animal study revealed decreased expression of UGP1A1 during pregnancy in rat liver [37]. Recent genome-wide association studies indicated the UGP1A1 were important genes in controlling serum bilirubin [35]. These results might partially explained the inconsistence between bilirubin concentration and ALT level in pregnant AHB patients.

The serum GGT levels were also significantly lower in pregnant patients. The serum GGT activity are lower in normal pregnant women than in non-pregnant women [36]. Hepatic synthesis of GGT could be inhibited by hormone secretion during pregnancy [36].

The strong point of the present study is the use of controls matched in age, sex, alcohol consumption, and other factors which may influence the course of AHB, with pregnant patients. Days from disease onset were also comparable between the two groups. To our knowledge, this is the first study that investigated the course of AHB during pregnancy with well matched controls.

There is a concern that 7 month follow up is too short. However, according to current acute hepatitis B investigative guidelines, those still HBV DNA-positive or HBsAg-positive are considered confirmed chronic carriers, and should be counseled accordingly [17]. In fact, we followed the patients for at least 1 year. For the pregnant AHB patients, none of them used antiviral therapy during follow-up, 4 patients were still HBsAg positive after at least 12 month follow-up. However, among the 618 AHB patients, for those without pregnancy, a significant portion of those still HBV DNA positive after 6 months of infection were treated with interferon or nucleotide analogues. Also, this was a hospital-based study, most patients in this study were symptomatic and relatively severe. The clinical features and outcome of asymptomatic AHB in pregnancy is not clear. To answer these questions, a large scaled population based study is needed.

## Conclusion

Although the clinical recovery of AHB differs little in pregnant and non-pregnant patients, the HBsAg loss and seroconversion is delayed and lower in pregnant patients. Pregnancy might be a possible risk of chronicity following acute HBV infection. Further clinical and basic studies are necessary to confirm the findings and to elucidate the underlying mechanisms.

## Abbreviations

AHB: Acute hepatitis B; ALT: Alanine aminotransferase; anti-HBc: Antibody to hepatitis core antigen; AST: Aspartate aminotransferase; GGT: Gamma-glutmyltransferase; HBsAg: Hepatitis B surface antigen; HBV: Hepatitis B virus.

## Competing interests

The authors declare that there are no competing interests.

## Authors’ contributions

YH: acquisition of data, technical support; CS: acquisition of data; CL: acquisition of data; SX: acquisition of data; DX: acquisition of data; LL: acquisition of data; JY: acquisition of data; WL: acquisition of data; QL: study concept and design, analysis and interpretation of data, drafting of the manuscript, study supervision. All authors read and approved the final manuscript.

## Pre-publication history

The pre-publication history for this paper can be accessed here:

http://www.biomedcentral.com/1471-2334/14/368/prepub
